# RECRUITMENT AND BASELINE RESULTS FOR THE STAR CAREGIVERS VIRTUAL TRAINING AND FOLLOW-UP PRAGMATIC TRIAL

**DOI:** 10.1093/geroni/igad104.2014

**Published:** 2023-12-21

**Authors:** Robert Penfold, Eric Johnson, Maggie Ramirez, James Ralston, Susan McCurry, Linda Teri

**Affiliations:** Kaiser Permanente Washington, Seattle, Washington, United States; KPWHRI, Seattle, Washington, United States; University of Washington School of Public Health, Seattle, Washington, United States; Kaiser Permanente Washington Health Research Institute, Seattle, Washington, United States; University of Washington, Seattle, Washington, United States; University of Washington, Seattle, Washington, United States

## Abstract

The STAR-Caregivers program is efficacious in reducing the behavioral and psychological symptoms of dementia (BPSD). In this pragmatic trial, we are testing a 6-session, self-directed, online version of the program that leverages secure email, an online learning management system, and brief coaching telephone calls to expand access to training. This paper reports on issues related to recruitment and baseline levels of psychosocial measures collected. Caregivers and persons living with dementia (PLWD) were randomized to intervention or usual care. Baseline measures were collected via REDCap and included standardized measures for PLWD dementia severity and functioning and caregiver measures for anxiety, stress, depression, and caregiving mastery. The study recruited 67 dyads. Recruitment increased greatly when we removed the inclusion that subjects be on antipsychotic medications. Baseline clinical showed the average score on the Dementia Severity Rating Scale was 22.2 (mild) and 28.8 on the Revised Memory and Problem Behavior Checklist (mild). The average Functional Activities Questionnaire was 22/30. Caregivers reported low levels of stress (KCSS), depression (PHQ8), and anxiety (GAD2) with mean and standard deviation: KCSS = 22.8 (6.6), PHQ8 = 4.9 (4.65), and GAD2 = 1.3 (1.4). The mean Caregiver Mastery Score was moderate at 20.4 (3.28). Virtual delivery of the STAR Caregiver training program is feasible. Caregivers of PLWD with mild to moderate cognitive and functional decline seem most amenable to intervention. We are currently analyzing outcome date to determine the efficacy of this program and plan to investigate the optimal timing of caregiver outreach and support programs.

